# Automatic Mode-Matching Method for MEMS Gyroscope Based on Fast Mode Reversal

**DOI:** 10.3390/mi16060704

**Published:** 2025-06-12

**Authors:** Feng Bu, Bo Fan, Rui Feng, Ming Zhou, Yiwang Wang

**Affiliations:** 1 School of Electronic and Information Engineering, Suzhou Vocational University, Suzhou 215104, China; 92010@jssvc.edu.cn (F.B.); wyiwang@163.com (Y.W.); 2 School of Electrical Engineering, University of South China, Hengyang 421001, China; 3 East China Institute of Photo-Electron IC, Suzhou 215163, China; richardfeng85@gmail.com (R.F.); zmlove08@163.com (M.Z.); 4 Suzhou Key Laboratory of Smart Energy Technology, Suzhou 215104, China

**Keywords:** MEMS disk resonator gyroscope, mode-matching, fast mode reversal, force to rebalance

## Abstract

Processing errors can result in an asymmetric stiffness distribution within a microelectromechanical system (MEMS) disk resonator gyroscope (DRG) and thereby cause a mode mismatch and reduce the mechanical sensitivity and closed-loop scale factor stability. This paper proposes an automatic mode-matching method that utilizes mode reversal to obtain the true resonant frequency of the operating state of a gyroscope for high-precision matching. This method constructs a gyroscope control system that contains a drive closed loop, sense force-to-rebalance (FTR) closed loop, and quadrature error correction closed loop. After the gyroscope was powered on and started up, the x- and y-axes were quickly switched to obtain the resonant frequencies of the two axes through a phase-locked loop (PLL), and the x-axis tuning voltage was automatically adjusted to match the two-axis frequency. The experimental results show that the method takes only 5 s to execute, the frequency matching accuracy reaches 0.01 Hz, the matching state can be maintained in the temperature range of −20 to 60 °C, and the fluctuation of the frequency split does not exceed 0.005 Hz.

## 1. Introduction

Microelectromechanical system (MEMS) vibrating gyroscopes have the advantages of small size, low weight, and low cost [1]. The axisymmetric structure helps to improve the energy transfer efficiency and vibration resistance of the gyro and has become an important candidate for high-performance micromechanical gyroscopes [2]. Mode–frequency matching between the drive and sense modes results in the maximum response gain and sensitivity [3,4]. Currently, the commonly used frequency tuning method is electrostatic tuning, which utilizes a negative stiffness electrostatic effect that is unique to the flat capacitor structure to change the modal frequency. This method can be divided into one-time mode matching and real-time mode matching.

One-time mode matching applies a fixed tuning voltage to the electrode. Typically, the resonant frequencies of the two modes are obtained via sweeping, and the tuning voltage is manually set. In addition, there are also automatic one-time matching methods. For example, Refs. [5,6] used the phase relationship between the input and output signals to achieve one-time automatic matching; however, this method occupies the Coriolis demodulation channel and can only be effective when there is no rotation rate input. Reference [7] uses the characteristic of the maximum amplitude of the response signal during mode matching; thus, it judges whether to match by detecting the amplitude of the quadrature error signal; however, the accuracy is low.

Real-time matching dynamically adjusts the mode frequency while the gyroscope operates. Currently, most methods require an injection of external excitation signals into the gyroscope to detect frequency split in real time by observing the amplitude [8,9] or phase [10,11] of the external force response. For example, Ref. [9] used the external force response amplitude in the sense mode to reflect the mode frequency split. This method severely limits the gyroscope bandwidth because it requires the application of a signal frequency that is outside the gyroscope bandwidth and cannot be offset significantly. Reference [10] proposed a real-time matching method using a virtual Coriolis force. This method injects a virtual Coriolis force into the sense mode and obtains the phase change in its response signal through phase-sensitive demodulation to characterize the mode frequency split. However, in such methods, the sense mode has vibration displacement caused by a non-external rotation rate, which is not allowed to be completely offset in closed-loop detection; therefore, it is not conducive to improving the robustness of the detection output to environmental changes.

Real-time matching methods without external signals have also been proposed. For example, Reference [12] leverages the power symmetry of both the noise and Coriolis response within the upper sideband (USB) and lower sideband (LSB) of the driving frequency. The power difference is considered a mismatch detection. However, this method results in large frequency fluctuations and low matching accuracy. References [13,14] used a double-sideband signal to excite a gyroscope and demodulate the amplitude of the double-sideband response to detect a frequency mismatch. However, the choice of double-sideband signal frequency has a significant impact on the matching accuracy. Moreover, some researchers have used neural network algorithms to achieve mode matching [15]; however, this method requires a large amount of data in advance to train the compensation model, which is impractical.

If the frequency split between the two modes remains unchanged or is within an acceptable range with environmental changes, one-time matching is the best solution. In this case, accurately obtaining the resonant frequency during gyroscope operation is the key to ensuring matching accuracy. The mode reversal technique is a method of switching control circuits to achieve the exchange of the drive and sense axis of a gyroscope, and this technique has been used for bias drift correction [16,17]. Accordingly, we designed an automatic mode-matching method based on mode reversal. During the closed-loop operation of the gyroscope, the resonant frequencies of the x- and y-axes are obtained by reversing the drive and sense modes in one cycle, and the tuning voltage is adjusted based on the frequency split to achieve one-time automatic matching. The entire matching process requires only 5 s, and the matching accuracy can reach within 0.01 Hz.

## 2. Impact of Frequency Spilt on FTR Closed-Loop Detection

### 2.1. Cobweb-like DRG

**Figure 1:**
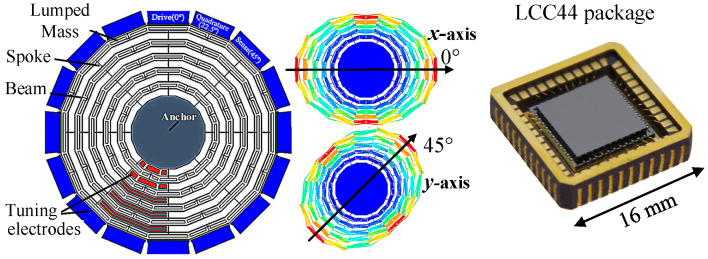
Structure of CDRG and LCC44 package.

A vacuum-packed cobweb-like disk-resonator gyroscope (CDRG) designed by Soochow University was used in the experiment [18]. Instead of a traditional ring structure, the CDRG implements a novel polygon structure that minimizes the structural symmetry error. Additionally, the initial frequency split is less than 2 Hz. The x-axis was oriented at 0° and 90°, and the y-axis was oriented at 45° and 135°. Furthermore, the maximum Q factor of the two modes was 180 k. Figure 1 illustrates the internal structure of the gyroscope and control circuit. Table 1 lists the gyroscope and circuit parameters used in this study.

### 2.2. FTR Closed-Loop System and Transfer Function

**Figure 2:**
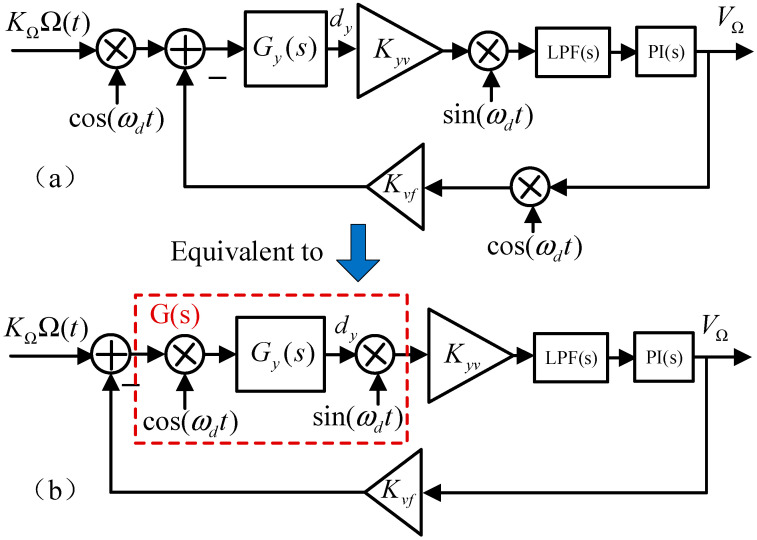
Simplified system diagram of Coriolis channel FTR. (**a**) The system diagram of the Coriolis-channel FTR. (**b**) Equivalent simplified system diagram.

Compared with the open-loop method, the FTR closed-loop detection method can extend bandwidth, increase range, and improve detection stability [19]. Figure 2a shows a system diagram of the Coriolis-channel FTR with the y-axis as the sense mode, where $V_{\Omega }$ is the amplitude of the feedback signal, $K_{yv}$ is the conversion coefficient from displacement to voltage in the signal pick-off circuit, $K_{vf}$ is the conversion coefficient from voltage to force, $K_{\Omega }$ is the conversion coefficient from the rotation rate to the Coriolis force ($K_{\Omega }=2m\omega_{d}\eta A_{x}$), and Ω(t) is the input rotation rate.

The input of the sense mode is the Coriolis force $K_{\Omega }\Omega (t)cos(\omega_{d}t)$ formed by the external rotation rate input. The output is the vibration displacement *d_y_*, which is converted into an electrical signal. The electrical signal is demodulated and filtered to obtain the vibration signal amplitude, which is input into the proportional integral (PI) controller to generate a feedback signal amplitude, denoted by $V_{\Omega }$. Then, $V_{\Omega }$ modulates the cosine signal, which is input into the sense mode to counteract the Coriolis force and thereby keep the sense mode relatively static.

Regarding the sense mode, the transfer function $G_{y}(s)$ is defined as

(1) $$G_{y}\left(s\right)=\frac{1/m}{s^{2}+\frac{\omega_{y}}{Q_{y}}s+\omega_{y}^{2}}$$

where $\omega_{y}$, $Q_{y}$, and m denote the natural resonant frequency, quality factor, and mass of the sense mode, respectively. For convenience, the modulation and demodulation processes are integrated with the transfer function $G_{y}(s)$ into transfer function G(s). The Coriolis FTR loop system can be simplified as shown in Figure 2b.

Moreover, combining $cos(\omega_{d}t)=\frac{\left(e^{j\omega_{d}t}+e^{-j\omega_{d}t}\right)}{2}$, $\sin(\omega_{d}t)=\frac{\left(e^{j\omega_{d}t}-e^{-j\omega_{d}t}\right)}{2j}$ with Equation (1) for the Laplace transform, the expression for G(s) can be obtained as follows:

(2) $$G\left(s\right)=\frac{j}{4}\left[G_{y}\left(s+j\omega_{d}\right)-G_{y}\left(s-j\omega_{d}\right)\right]\;=\frac{1}{2m}\frac{2\omega_{d}s+\frac{\omega_{y}\omega_{d}}{Q_{y}}}{{\left(s^{2}+\frac{\omega_{y}}{Q_{y}}s+\omega_{y}^{2}-\omega_{d}^{2}\right)}^{2}+{\left(2\omega_{d}s+\frac{\omega_{y}\omega_{d}}{Q_{y}}\right)}^{2}}$$

Furthermore, PI(s) and LPF(s) are the transfer functions of the PI controller and LPF, respectively, and are expressed as

(3) $$PI(s)=K_{P}+\frac{K_{I}}{s}$$

(4) $$LPF(s)=\frac{\omega_{c}}{s+\omega_{c}}$$

where $K_{P}$ and $K_{I}$ represent the proportional and integral coefficients of the PI controller, respectively, and $\omega_{c}$ represents the cutoff frequency of the LPF.

According to Figure 2b, the transfer function of the FTR closed-loop system can be obtained as [20,21]

(5) $$H_{close}\left(s\right)=\frac{V_{\Omega }\left(s\right)}{\Omega \left(s\right)}=\frac{K_{\Omega }K_{yv}G(s)LPF(s)PI(s)}{1+K_{vf}K_{yv}G(s)LPF(s)PI(s)}$$

The transfer functions of the FTR closed-loop system and submodules are expressed. However, it is difficult to include these submodules in $H_{close}\left(s\right)$ for further calculations, particularly for high-order transfer functions G(s). Therefore, it is necessary to reduce the order of G(s).

The following two reasons are considered: (a) the fourth-order coefficient, which is equal to 1, and the third-order coefficient, which is equal to $\frac{2\omega_{y}}{Q_{y}}$, are small compared with other order coefficients; (b) the high-order term only affects the high-frequency response of the system (above kHz), whereas the gyroscope bandwidth is usually low (below 100 Hz). In addition, we find that two non-dominant poles of G(s) are far away from the origin and have little influence on the system. Hence, these poles can be omitted. Therefore, the fourth-order system G(s) can be simplified as a second-order system that does not affect the frequency response characteristics of the entire system.

(6) $$G(s)\approx \frac{1}{2m}\frac{2\omega_{d}s+\frac{\omega_{y}\omega_{d}}{Q_{y}}}{s^{2}\left(\frac{\omega_{y}^{2}}{Q_{y}^{2}}+2(\omega_{y}^{2}+\omega_{d}^{2})\right)+s\left(\frac{2\omega_{y}^{3}+2\omega_{y}\omega_{d}^{2}}{Q_{y}}\right)+(\omega_{y}^{2}-\omega_{d}^{2}{)}^{2}+\frac{\omega_{y}^{2}\omega_{d}^{2}}{Q_{y}^{2}}}$$

In the case of a small frequency split, the FTR can make the frequencies of the two modes approximately equal, and this technique further simplifies the system without affecting the analysis of the closed-loop characteristics. This assumption holds for three reasons: (1) for the current popular axisymmetric gyroscopes, the original frequency split is usually small, (2) the frequency split can be reduced to a significantly small range (usually less than 1 Hz) using electrostatic tuning technology, and (3) FTR closed-loop control can suppress the influence of frequency mismatch to ensure that the effect of a small frequency split on bandwidth and noise can be ignored.

Considering the two conditions $\omega_{y}\approx \omega_{d}$ and $1/Q_{y}^{2}<<1$, G(s) can be simplified as follows:

(7) $$G(s)\approx \frac{1}{2m\omega_{y}}\frac{1}{2s+\frac{\omega_{y}}{Q_{y}}}$$

### 2.3. Impact of Frequency Spilt on System Response

**Figure 3:**
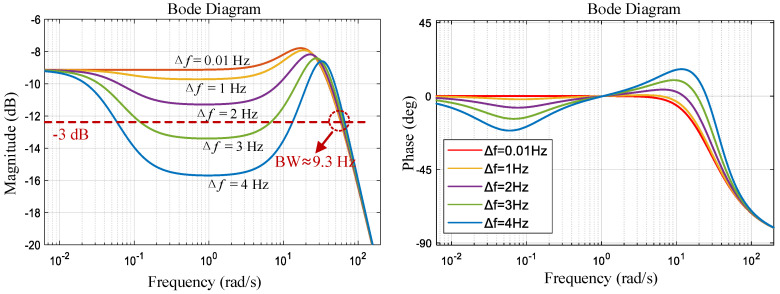
Frequency response curves of $H_{close}\left(s\right)$ at different frequency splits (theoretical calculations).

The frequency response curves obtained using MATLAB 2023b tools are shown in Figure 3 at different frequency splits ($\Delta f=\frac{\left(\omega_{x}-\omega_{y}\right)}{2\pi }$). The Δ*f* term significantly affects the passband flatness (i.e., the stability of the scale factor). A larger value of Δ*f* implies a more pronounced phenomenon of passband depression, which affects the stability of the scale factor. When Δ*f* > 2 Hz, the passband dip reaches −3 dB, which reduces the bandwidth. Therefore, frequency matching can improve the stability of the gyroscope scale factor and bandwidth.

The passband flatness and closed-loop bandwidth are affected by the closed-loop control gain $K_{vf}K_{yv}$. When the gain increased, the FTR had a stronger ability to suppress the effect of the frequency split. For gyroscopic devices with large frequency splits, it is necessary to significantly increase the control gain to control the scale factor error within a specified range, although a large control gain deteriorates the noise performance of the gyroscope [22]. This tradeoff can be effectively solved via mode tuning.

## 3. Automatic Mode-Matching Based on Mode Reversal

**Figure 4:**
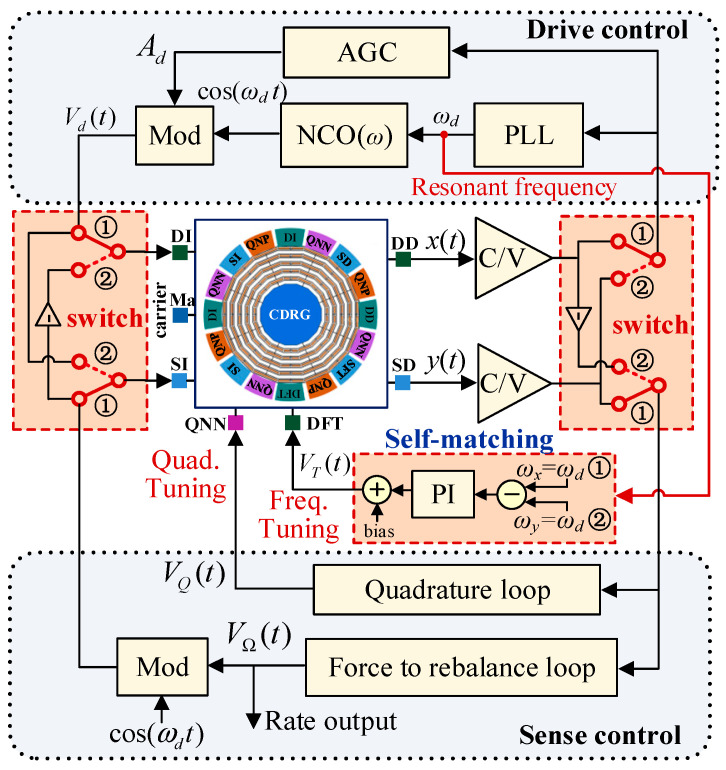
Control system diagram with a mode reversal frequency self-matching module.

The gyroscope control system adopts the electromechanical amplitude modulation (EAM) signal detection method [23], which applies high-frequency carriers to the gyroscope mass to modulate the amplitude of the vibration signal and suppress the feedthrough interference. The sense-mode closed loop consists of a Coriolis FTR loop and quadrature stiffness control loop, as shown in Figure 4. The FTR loop generates a feedback signal based on the amplitude of the Coriolis response, which is fed into the sense mode to counteract the in-phase coupling and Coriolis forces. The output of the PI controller was the rotation rate detection output. The quadrature loop generates a DC voltage based on the quadrature coupling response amplitude and applies it to the quadrature tuning electrode for stiffness correction.

**Figure 5:**
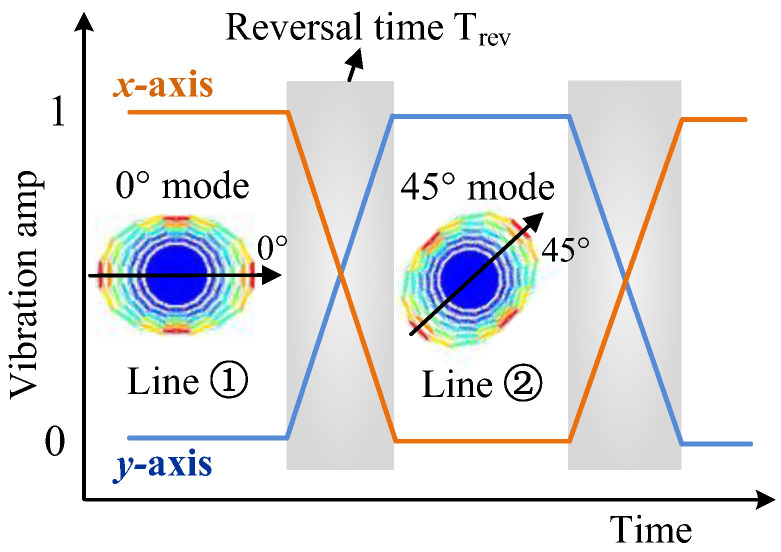
Working principle of the mode reversal scheme.

By setting two electronic toggle switches, the input and output of the x- and y-axes can be switched in the working state of the gyroscope. When switching to line ➀, the x-axis is the drive mode, and the y-axis is the sense mode, defined as the 0° mode. When switching to line ➁, the y-axis is the drive mode and the x-axis is the sense mode, defined as the 45 ° mode, as shown in Figure 5.

The output of the phase-locked loop (PLL) module is the frequency $\omega_{d}$ of the drive-mode excitation signal, which is $\omega_{d}=\omega_{x}$ or $\omega_{y}$, in the resonant state. The resonant frequencies $\omega_{x}$ and $\omega_{y}$ of the x- and y-axes can be obtained by reversing the mode in one cycle. Then, using the PI controller, the frequency-tuning voltage $V_{T}$ of the x-axis is adjusted to make $\omega_{x}=\omega_{y}$ and lock the tuning voltage to remain unchanged.

## 4. Experiment and Discussion

### 4.1. Experimental Platform

**Figure 6:**
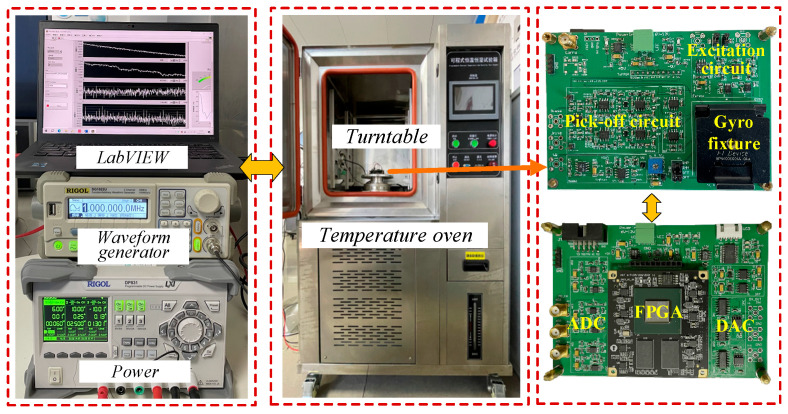
Control circuit and experimental setups.

A Xilinx artix-7 series FPGA (Xilinx, San Jose, CA, USA) was used as the gyroscope digital control system. Relevant data were collected using serial ports and LabVIEW 2024 software at a sampling rate of 100 Sa/s. In addition, the carrier applied to the mass block was a sine signal with a peak value of 5 V and a frequency of 1 MHz. The control circuit of the CDRG and the experimental environment are shown in Figure 6.

### 4.2. Response Testing at Different Frequency Splits

To overcome the limitations of the frequency sweep function of the rate table, we used the virtual Coriolis force method proposed by Peking University to measure the gyroscope bandwidth [24]. In this method, a variable-frequency virtual-rate signal is first generated and modulated using a reference signal of the same frequency and phase as the real Coriolis force. Subsequently, the signal was inputted to the excitation end of the gyroscope sense mode, which was simulated as the input for the external Coriolis force.

**Figure 7:**
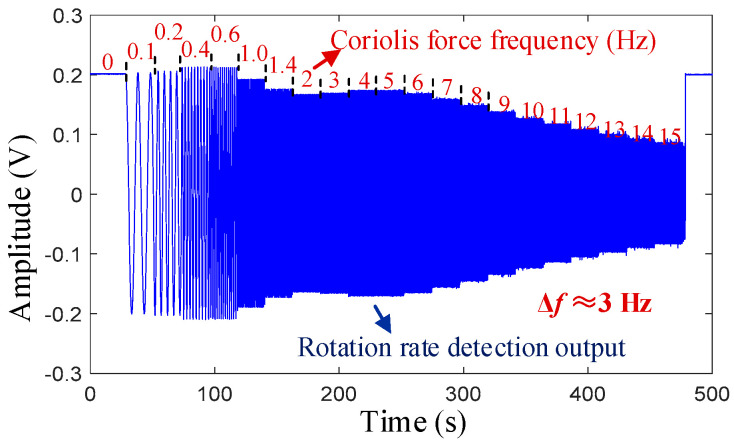
Frequency response output at Δ*f* ≈ 3 Hz.

In the 0° mode, the amplitude of the virtual rate is set as $\Omega_{0}$ = 35 °/s (corresponding to a voltage output value of approximately 210 mV), and the frequency $f_{\Omega }$ gradually increases from 0.1 to 15 Hz, with each frequency point being maintained for 20 s. Figure 7 shows the rate detection output signal at a frequency split of Δ*f* ≈ 3 Hz in the frequency response test.

**Figure 8:**
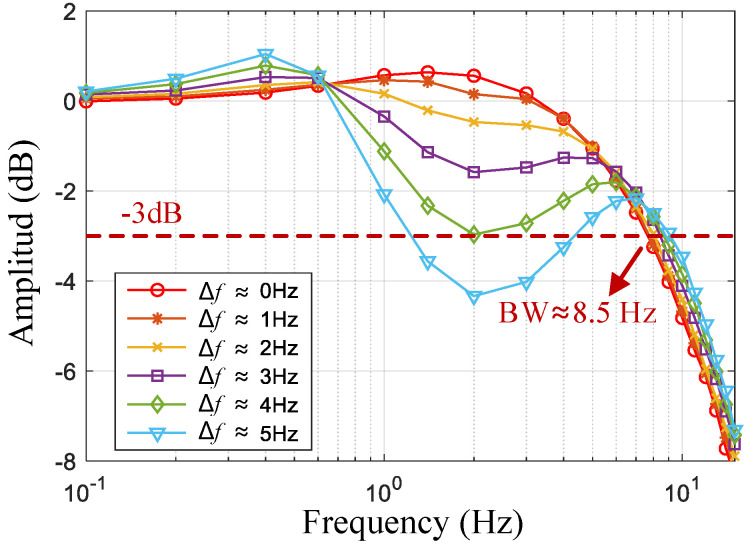
Frequency response curves of a gyroscope at different frequency splits (test values).

By adjusting the tuning voltage of the x-axis to change the resonant frequency, the variation in Δ*f* from 0 to 5 Hz can be achieved. Figure 8 shows the frequency response curves at different frequency splits. When the Δ*f* is small, the response curve within the passband is stable with a bandwidth of approximately 8.5 Hz. As Δ*f* increases, the concave phenomenon of the frequency response curve becomes more pronounced. These results are consistent with the theoretical calculation results shown in Figure 3.

### 4.3. Frequency Self-Matching Test

**Figure 9:**
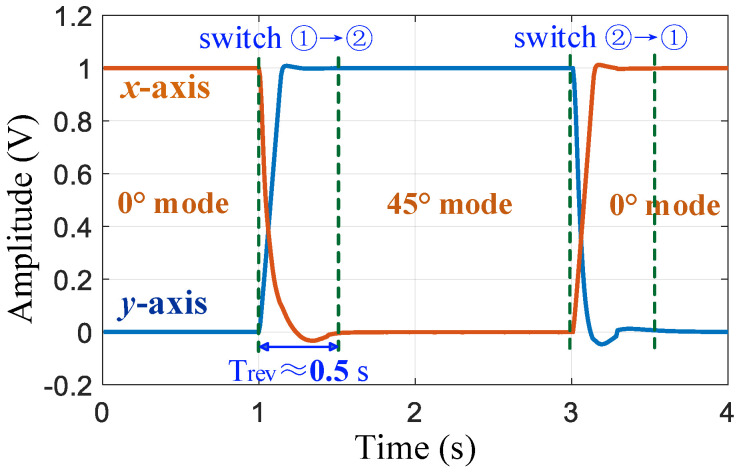
Changes in amplitude of the x-axis and y-axis vibration signals during the mode reversal process.

**Figure 10:**
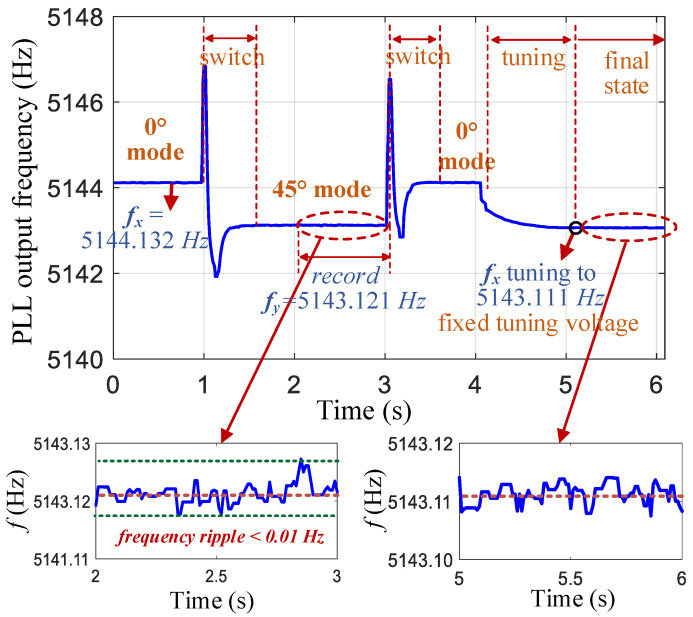
PLL output frequency curve during mode reversal and the frequency self-matching process.

After the gyroscope is powered on at room temperature, it automatically performs mode reversal and frequency self-matching operations. First, the 0° mode is switched to the 45° mode. After stabilization, the mode is switched back to 0°. The amplitude changes in the x- and y-axis vibration signals are shown in Figure 9. During the stability period of the 45° mode, the resonant frequency of the y-axis is recorded (this frequency value was the average of data within 1 s). After the mode is switched back to the 0° mode and stabilized, the tuning voltage *V_T_* of the x-axis is adjusted to 5.82 V so that $f_{x}$ decreases from 5144.13 to 5143.11 Hz to achieve frequency matching. The frequency curve of the PLL output during this process is illustrated in Figure 10. The entire automatic matching process required less than 5 s, with the mode-reversal process taking only 0.5 s to complete. After matching, the mode–frequency split was reduced to within 0.01 Hz.

The resonant frequency is output by the PLL in the FPGA-based control system with a data bit size of 30 bits, including 20 decimal places, and a frequency resolution of $10^{-6}$ Hz. Owing to the presence of circuit and gyroscope noises, the frequency output data contain random noise. Figure 10 shows that the ripple of the frequency data was within 0.01 Hz. In addition, the frequency-tuning circuit adopted a high-resolution (16 bit) and low-noise (10 nV/$\sqrt{Hz}$) ADC. All these factors contributed to the tuning accuracy reaching 0.01 Hz.

### 4.4. Temperature Testing

**Figure 11:**
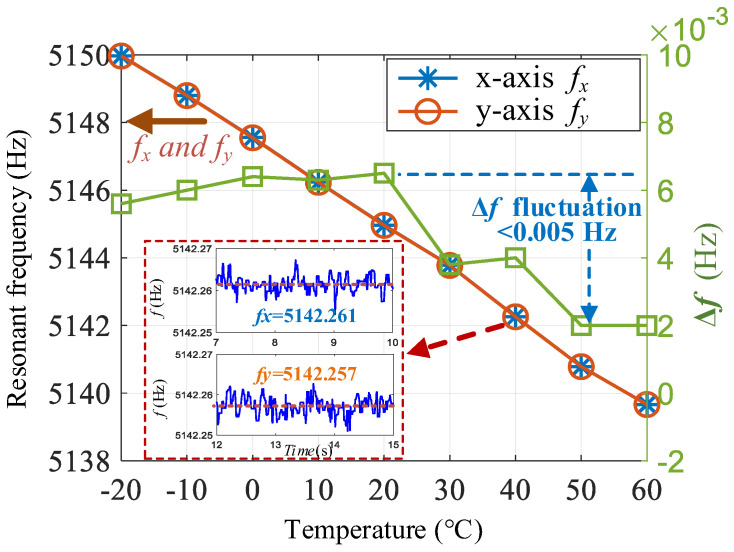
Variation curve of the frequency split with temperature after one-time automatic matching.

To verify the effectiveness of one-time mode matching, automatic mode matching was performed at room temperature to obtain a fixed tuning voltage *V_T_*. Then, the temperature of the temperature oven was increased from −20 to 60 °C with a temperature step of 10 °C and a dwell time of 20 min at each temperature point. Figure 11 shows the variation curves of the resonant frequencies along the two axes. The frequencies of both axes decrease with an increasing temperature. When the temperature rises by 80 °C, the frequency decreases by 10.3 Hz, and the temperature sensitivity of the frequency is –0.129 Hz/℃.

Because the same mass block is used for the x- and y-axes in the axisymmetric disk resonant gyroscope, the frequency split is relatively stable. Within the range of 80 °C, the maximum fluctuation of Δ*f* is only 0.005 Hz. Hence, after one-time mode-matching, the mode-matching state can be maintained when the ambient temperature changes. In addition, because the FTR closed-loop detection system itself suppresses the adverse effects of frequency mismatch, a small frequency split does not have a significant impact on the detection output. Therefore, one-time automatic matching can achieve a good performance without the need for real-time matching.

## 5. Conclusions

The performance of a fully symmetrical gyroscope is affected by the mode–frequency split. When the gyroscope frequency split is relatively stable under environmental changes, one-time matching is the simplest and most effective method. To overcome the problem of the resonant frequency obtained using traditional mode-matching methods not being the true frequency in the working state, an automatic mode-matching method based on mode reversal is proposed. A gyroscope control circuit that included a force to rebalance the loop and a quadrature correction loop was designed. When the gyroscope was powered on, the x- and y-axes were switched to obtain the accurate resonant frequencies of the two axes and achieve automatic matching. The test results show that automatic matching can achieve a frequency split of less than 0.01 Hz, and the frequency split fluctuation does not exceed 0.005 Hz within the temperature range of −20 to 60 °C. This matching state was maintained over a wide range of temperatures.

Table 2 summarizes mode-matching techniques based on the electrostatic negative-stiffness effect published in recent years. The matching accuracy achieved by the proposed mode-matching method is superior to those of the most existing mode-matching methods.

However, the proposed method is only performed when the gyroscope is powered on because the mode reversal process needs to be performed when the gyroscope is stationary; otherwise, the detection of the rotation rate will be affected. To solve the problem of frequency split deterioration after long-term operation and achieve in-run matching, in future work, we will utilize the gap time (a few seconds) during the temporary stationary state of the gyroscope to trigger a self-matching process based on mode reversal. The temporary static state is determined by detecting the vibration of the gyroscope carrier using a high-sensitivity three-axis accelerometer.

## Figures and Tables

**Table 1:** Basic parameters of the gyroscope and circuit.

Parameters	Values
Proof mass (*m*)	0.46 mg
Parallel plate gap (*g*)	7 um
Parallel plate capacitance (*C*_0_)	2 pF
*x*-axis resonant frequency (*f_x_*)	5144.3 Hz
*y*-axis resonant frequency (*f_y_*)	5143.1 Hz
*x*-axis Q-factor (*Q_x_*)	181.2 k
*y*-axis Q-factor (*Q_y_*)	184.5 k
Carrier signal	5V_pk_@1MHz
Scale factor (*SF*)	6.1 mV/°/s

**Table 2:** Summary of mode-matching techniques.

Research Institution	Gyroscope	Tuning Accuracy	Technical Characteristics
Middle East Technical University [5]	Double-mass gyroscope	<1 Hz	Using phase between the residual quadrature and drive signals, one-time
Nanjing University of Science and Technology [10]	Disk resonator gyroscope	0.094 Hz	Using phase-shifted virtual Coriolis force, real-time
Southeast University [11]	Disk resonator gyroscope	<0.1 Hz	Using virtual Coriolis force, one-time
Southeast University [12]	Double-mass gyroscope	0.28 Hz	Using power symmetry of readout signal, real-time
Newcastle University [22,25]	Ring gyroscope	0.006 Hz	Using response of an external force, one-time
University of California at Los Angeles [26]	Ring gyroscope	<0.08 Hz	Using mass perturbation, parametric resonator models
Proposed method	Cobweb-like disk resonator gyroscope	0.01 Hz	Using fast mode reversal, one-time

## Data Availability

The original contributions presented in the study are included in the article, further inquiries can be directed to the corresponding author.
